# The effect of fibrin glue in preventing staple-line leak after sleeve gastrectomy. An experimental study in rats ^1^

**DOI:** 10.1590/s0102-865020190080000001

**Published:** 2019-10-14

**Authors:** Yasar Ozdenkaya, Oktay Olmuscelik, Pelin Basim, Burcu Saka, Naciye Cigdem Arslan

**Affiliations:** I MD, Assistant Professor, Department of General Surgery , Medipol University Medical Faculty , Istanbul , Turkey . Conception and design of the study, acquisition of data, manuscript writing.; II MD, Assistant Professor, Department of Internal Medicine , Medipol University Medical Faculty, Istanbul , Turkey . Conception of the study, acquisition of data, critical revision.; III MD, Assistant Professor, Department of General Surgery , Medipol University Medical Faculty , Istanbul , Turkey . Conception and design of the study, acquisition of data, critical revision.; IV MD, Assistant Professor, Department of Pathology , Medipol University Medical Faculty , Istanbul , Turkey . Histopathological examinations.; V MD, Associate Professor, Department of General Surgery , Medipol University Medical Faculty , Istanbul , Turkey . Statistics analysis, manuscript writing, final approval.

**Keywords:** Gastrectomy, Fibrin Tissue Adhesive, Rats

## Abstract

**Purpose:**

To evaluate the effect of fibrin glue on staple-line leak after sleeve gastrectomy.

**Methods:**

Fourteen adult wistar rats 300 gr were randomized into two groups: Control group (n=7) and study group (n=7). All the rats underwent sleeve gastrectomy using lineer stapler. In the study group, fibrin glue was used to reinforce the staple-line. The rats were sacrificed 7 days after surgery. The stomach was resected, submerged in saline and exposed to excess pressure to obtain a burst pressure value. The gastric staple line was evaluated histopathologically according to the Ehrlich Hunt scale. The results of the two groups were compared.

**Results:**

The mean Ehrlich-Hunt scores for inflammation, fibroblastic activity and neo-angiogenesis were similar between the groups (p>0.05). Collagen deposition was significantly higher in study group (3.42±0.53) when compared with control group (2.57±0.78) (p=0.035). The mean burst pressure was 137.8±8.5 mmHg for control group and 135.0±8.1 mmHg for study group (p=0.536).

**Conclusion:**

Reinforcement of the staple-line with fibrin glue has no effect on the burst pressure after sleeve gastrectomy. More studies are needed to evaluate the precautions against leak after sleeve gastrectomy.

## Introduction

Sleeve gastrectomy (SG) is a primary bariatric procedure which has been used increasingly during recent years. Technichal simplicity, documented positive metabolic benefits and effective weight loss have contributed to the popularity of this procedure. Currently, SG comprises more than half of the bariatric procedures in some parts of the USA and Europe ^1^ . Leak from the staple-line is the main challenging complication after SG; with reported rate of up to 7% ^2^ . Reinforcement methods including oversewing and application of several sealants have been preferred by most of the surgeons; however, there is still no consensus on neither the effectiveness nor the best method of reinforcement. Most of the studies showed that reinforcement of the staple-line does not reduce the leak rate ^3 - 5^ . On the contrary, the benefits of bioabsorbable sealents have been reported in wide series ^6 - 8^ .

Fibrin glue (FG) is designed to mimic the final steps of the blood coagulation cascade, forming a stable physiological fibrin clot that assists hemostasis and wound healing. Fibrin tissue adhesive initiates the final phase of physiologic clot formation, thus contributing to tissue healing by increased proliferation of fibroblasts. Benefit and safety of FG on several surgical procedures including intestinal anastomoses, cardiovascular surgery, liver and spleen trauma, dural tear and bronchial fistulas have been reported ^9 - 12^ .

The conflicting results of the studies raise the need for more research for the effect of FG on staple-line leak after SG. The aim of this experimental study was to investigate whether FG is an effective reinforcement method to prevent leak after SG in rats.

## Methods

This study was conducted at an Institutional Experimental Animals Research Unit with the approval of the Experimental Animals Ethics Committee. All the animal experiments were conducted in conformity with the International Guiding Principles for Biomedical Research Involving Animals.

Fourteen Wistar albino rats with an average weight of 300 gr were used in the study. The animals were kept in stainless steel cages under standard conditions (temperature 20±2˚Ca, humidity 45%, 12 hours daylight/dark cycle) and were fed ad libitum with normal pellet. All the rats were acclimatized before the experiment.

A commercially available steam-sterilized, 2-component fibrin tissue adhesive (Tisseel ^®^ , Baxter International, Deerfield, Illinois, USA) was used. It consists of four vials, two of which are lyophilized (adhesive protein concentrate and thrombin) and the other two are diluents (fibrinoysis inhibitor solution and calcium chloride solution). Vials 1, 2 and 4 are obtained from pooledhuman plasma and vial 2 is of bovine-origin.

### Surgical procedure 

**Figure 1:**
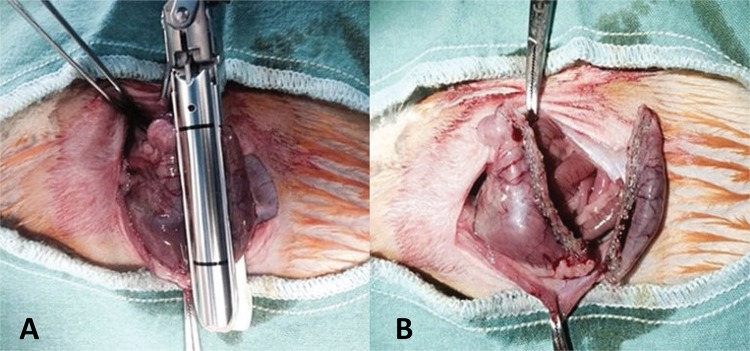
A. Sleeve gastrectomy with linear stapler. B . Intraoperative view of stapler line after resection.

Intramuscular 5 mg/kg xylazine hydrochloride (Rompun ^®^ , 2% injectable solution 20 mg/mL, Bayer, Canada) and 20 mg/kg ketamine hydrochloride (Ketalar ^®^ , injectable solution 100 mg/ml Pfizer, UK) were administered. The abdomen was shaved and cleaned with betadine prior to surgery. All the rats underwent SG by the same surgeon in the same fashion. The rats were placed in supine position on the operating table. A 4 cm midline incision was made. Stomach was identified, and gastrosplenic ligament was ligated with 4/0 silk sutures and divided. The great omentum was ligated with 4/0 silk sutures and divided down to the level of pylorus. The transection of the stomach was started 3-5 mm above the pylorus and a part of the pylorus was left intact to maintain the passage to the duodenum. The transection line was marked by stitches which suspend gastrics fundus. A lineer stapler (Covidien Endo GIA ^™^ , EGIA30CTAVM white cartridge) was positioned on the marked line and the great curvature along with the gastric fundus was removed ( Fig. 1 ). Orogastric tube was not used during the resection. Differently from the control group, FG (Tissel ^®^ , Baxter AG, Vienna, Austria) was applied on the stapler line following resection in the study group. Finally, the abdominal wall was closed with running 3/0 polyglycolic suture, and skin was closed with 4/0 running intacutaneus suture to prevent the stitches to be eaten by the rats.

### Evaluation of leak 

After surgery, the rats were allowed to take water on the first post-operative day and normal pellet ad libitum on the second day. Sacrification was performed by CO₂ inhalation on postoperative day 7. The skin was opened, and the sleeve gastrectomy site was explored in terms of mucosal discontinuity, presence of food residue and secretions. Staple-line leak was described as any opening on staple-line, food residue or purulent drainage in the abdomen.

### Measurement of burst pressure 

An analog manometer (Perfect Aneroid ^™^ 40 mm, Erka, Germany) was used to observe the intragastric pressure. After intraabdominal exploration, gastrectomy with subtotal esophagectomy was performed. A 16G catether was inserted through the pylor and secured with a suture. Another catheter was inserted through the esophagus and connected to the manometer. The entire system was submerged, and continuous intraluminal pressure was monitored during the air insufflation with the pumping device. The pressure level of the manometer was recorded as the burst pressure when the first air bubble was seen in the staple-line.

### Assesment of histopathologic changes 

The gastrectomy suture line was excised and fixed in 10% formalin solution for pathological evaluation. Parrafin sections were prepared with Hematoxylin and eosin, Masson trichrome and CD31 staining. The specimens were evaluated in terms of inflammatory cell infiltration, fibroblastic activity, collagen deposition and neo-angiogenesis by using modified Ehrlich-Hunt scale as follows; 0: no evidence, 1: little, but scattered, 2: small amounts and in every field, 3: large amounts, but scattered, 4: large amounts and present at every area ^13^ . All the specimens were assessed by two pathologists who were unaware of the groups.

### Statistical analysis 

The data were analyzed by SPSS for Windows V.20.0 (SPSS Inc, Chicago, IL). The variables were expressed as the mean±standard deviation (SD). The independent *t* test was used to analyze the difference between the histpathological score and burst pressure of the groups. p<0.05 was considered statistically significant.

## Results

Appropriate staple closure was achieved in all the surgical procedures. There were no technical or surgical complications such as mechanical stapling mistake or hematoma formation in the staple line. The resected specimens or intraabdominal findings showed no evidence for leak. Details of the results of both groups are given in Table 1 .

**Table 1:** Details of histopathologic examination and burst pressure in rats.

	Rats (n=22)	Inflammation	Fibroblastic activity	Neo-angiogenesis	Collagen deposition	Burst pressure (mmHg)
Control group (n=7)	C1	1	2	2	1	140
	C2	4	3	2	2	140
	C3	3	4	3	3	135
	C4	3	3	3	3	145
	C5	3	3	3	3	120
	C6	3	2	1	3	140
	C7	2	2	2	3	145
Study group (n=7)	S1	2	3	3	4	145
	S2	3	3	3	4	145
	S3	2	2	2	3	135
	S4	2	3	2	3	135
	S5	4	4	3	4	125
	S6	2	3	2	3	125
	S7	4	4	4	3	135

According to the Ehrlich Hunt scale, significant differences were found between the groups only in terms of collagen deposition; the study group had significantly higher mean score (3.42±0.53) than the control group (2.57±0.78) (p=0.035). Inflammation, fibroblastic activity and neo-angiogenesis scores were similar (p>0.05). The mean burst pressures of staple line in the control group (137.8±8.5) and the study group (135±8.1) were not significantly different (p=0.536) ( Table 2 ).

**Table 2:** Comparison of control and study groups.

	Control group (n=7)	Study group (n=7)	p
Inflammation (mean±SD)	2.71±0.95	2.71±0.95	1.000
Fibroblastic activity (mean±SD)	2.71±0.75	3.14±0.69	0.290
Neo-angiogenesis (mean±SD)	2.28±0.75	2.71±0.75	0.310
Collagen deposition (mean±SD)	2.57±0.78	3.42±0.53	0.035
Burst pressure (mmHg, mean±SD)	137.8±8.5	135±8.1	0.536

## Discussion

The risk factors for leak after SG includes male gender, higher body mass index, concomitant sleep apnea, use of small bougies, the type of staple cartridges, open surgery, prolonged operative time, the occurrence of intraoperative complications and inadequate learning curve ^14 - 16^ . During the last decade, preventing staple-line leak has been the main issue among bariatric surgeons. Despite there is no clear evidence, in a survey at the Fourth International Consensus Summit on Sleeve Gastrectomy, 75% of the surgeons claimed that strengthening the staple-line may prevent postoperative bleeding and leak after SG ^17^ . A variety of reinforcement methods including oversewing, peritonealization, omental wrap, buttressing with bioabsorbable materials, non-absorbable strips, and sealant agents have been described for this purpose ^18^ .

In their meta-analysis of 8 randomized trials and 453 patients comparing reinforcement methods with no reinforcement, Wang *et al* . ^18^ concluded that using buttressing or roofing materials reduced postoperative bleeding and overall complications but had no effect on staple-line leaks. Moreover, overwesing had no benefit and resulted in longer operative time. In this meta-analysis there were 3 studies with 117 patients comparing FG and no reinforcement ^19 - 21^ . There was no difference in leak rates of two groups (p=0.365). In another study, Carandina *et al* . ^5^ randomized 600 patients into no reinforcement, oversewing and FG groups. Mean operative time was shorter in the FG group when compared with oversewing (p<0.0001). Postoperative bleeding, leak and stenosis were similar between 3 groups. Oversewing seems to be burdened by unnecessary costs, more complicated procedure, prolonged operative time and increased stenosis rate ^4^ . In a systematic review, Gagner *et al* . ^8^ reported that only absorbable polymers were associated with decreased rate of leak (1.01%) when compared with oversewing, non-absorbable porcine strips and no reinforcement. These results have led the surgeons to focus on sealent agents rather than on suturing the staple-line.

Fibgrin glue is a widely used and safe absorbable material that provides hemostasis, sealing and adhesions ^22^ . The safety and feasibility of FG in laparoscopic bariatric procedures were reported in several studies ^23 , 24^ . However, most of these studies include different bariatric procedures other than SG and report retrospective results. Coskun *et al* . ^7^ reported no leak after 1000 laparoscopic SGs with FG application. They concluded that spraying FG on staple line and left diaphragma helps hemostasis and keeps the normal anatomical position of the stomach which may prevent stricture, twist and leak in the staple line. In further clinical studies comparing FG and other reinforcement methods, no clear benefit of SG in preventing leak has been showed ^5 , 6^ .

Most of the ex-vivo studies on SG in the literature have examined the resected human specimens. There are only a few experimental animal studies. Conformably with clinical studies, the results of ex-vivo and experimental studies showed no benefit of buttressing agents in preventing staple-line leak. In a randomized-controlled study, Konca *et al* . ^25^ tiered 32 rats into 4 groups: 12-Fr bougie, 8-Fr bougie, 12-F bougie and FG, 8-F bougie and FG. They assessed burst pressure, staple-line leak, tissue oxygen partial pressure and hydroxyproline levels at esophagogastric junction between groups. There was no difference in terms of burst pressures, tissue oxygen partial pressure and tissue hydroxyproline levels. The only rat with mortality from staple-line leak was in the 8-Fr Bougie group. One of the limitations of our study was not using an orogastric tube or bougie. Another difference with human studies was using a single white cartridge which minimizes the risk of overlapping sequential staples and associated leak. In one study, Bakers *et al* . ^26^ demonstrated a burst pressure of 140 mmHg in rats which was quite higher when compared to 60 mmHg pressure in porcine stomach. The white cartridge is one of the smallest stapler devices available; however, we consider that the thickness and burst pressure of the rat stomach was compatible with the staple thickness. According to Laplace`s in cylinders of different diameters and the same internal pressure, a tension on the walls will be greater for the larger the diameter. In order to ensure survival until sacrification, our rats did not undergo stapling very close to the esophagus, the angular incisura and pylorus which were in favor of leaks. However, the staple line was very short when compared with human SG and this might decrease the risk of undetected small defects. In brief, assessing human SG in a rat model has several conflictions and bias.

In our study none of the rats had leak. The only parameter which was influenced by FG was increased tissue collagen levels. In another experimental study of 18 pigs, bovine pericardium was used as a buttressing agent. Aydin *et al* . ^27^ compared the burst pressures on resected specimens and the postoperative complications of 118 patients with no reinforcement, oversewing, FG application and bioabsorbable strips. They found that oversewing provided the highest burst pressure but there was no correlation between leak and burst pressures. Karakoyun *et al.* ^28^ compared the burst pressures on resected specimens in patients that underwent no reinforcement, oversewing and FG. They showed higher burst pressure in specimens with owersewing (p=<0.01).

Consistently with the literature, our results showed that FG had no effect on burst pressure; however, the effect on staple-line leak is difficult to speculate. There is no evidence that burst pressure represents staple-line leak in sleeve gastrectomy. Moreover, the time until sacrification and the number of experimental subjects were not sufficient to evaluate staple-line leak in our study as well as in the previous studies. Gastric leak after bariatric surgery is observed more frequently on the 10th postoperative day, but may occur during the first 30 postoperative days ^29^ . Apart from these, the limited number of the subjects and lack of analysis regarding operative time and bleeding were the main drawbacks of our study.

Although there is no clear evidence indicating the favourable effects, FG has still been widely used in bariatric procedures. Our results revealed that FG application on the staple-line leads to favorable histophatologic changes compared with no reinforcement. However, these changes did not seem to reflect in a significant increase in burst pressure and decrease in leak rate. Further prospective randomized experimental and clinical studies are needed to clarify the effect of reinforcement methods in SG.

## Conclusion

Reinforcement of the staple-line with fibrin glue has no effect on the burst pressure after sleeve gastrectomy.

## Footnotes

1 The research was performed in Istanbul Medipol University Animal Experiments Laboratory, Turkey.
